# Supplementary material for: Resolving the full spectrum of human genome variation using Linked-Reads
Source: Genome Res. 2019 Apr;29(4):635–45. doi: 10.1101/gr.234443.118 (PMC6442396; doi:10.1101/gr.234443.118)
Supplement: Supplemental Material [file supp_gr.234443.118_Supplemental_longranger-2.2.zip › longranger-2.2.tar.bz2!/lr2.2-public/lib/stan/cmdstan/stan/doc/api/html/classstan_1_1mcmc_1_1stepsize__adaptation-members.html]

Stan: Member List


|  |  |
| --- | --- |
|  | Stan  2.5.0  probability, sampling & optimization |


- Main Page
- Related Pages
- Namespaces
- Classes
- Files

- Class List
- Class Index
- Class Hierarchy
- Class Members

All Classes Namespaces Files Functions Variables Typedefs Enumerations Enumerator Friends Macros Pages

- stan
- mcmc
- stepsize\_adaptation

stan::mcmc::stepsize\_adaptation Member List

This is the complete list of members for stan::mcmc::stepsize\_adaptation, including all inherited members.

|  |  |  |
| --- | --- | --- |
| complete\_adaptation(double &epsilon) | stan::mcmc::stepsize\_adaptation | inline |
| counter\_ | stan::mcmc::stepsize\_adaptation | protected |
| delta\_ | stan::mcmc::stepsize\_adaptation | protected |
| gamma\_ | stan::mcmc::stepsize\_adaptation | protected |
| get\_delta() | stan::mcmc::stepsize\_adaptation | inline |
| get\_gamma() | stan::mcmc::stepsize\_adaptation | inline |
| get\_kappa() | stan::mcmc::stepsize\_adaptation | inline |
| get\_mu() | stan::mcmc::stepsize\_adaptation | inline |
| get\_t0() | stan::mcmc::stepsize\_adaptation | inline |
| kappa\_ | stan::mcmc::stepsize\_adaptation | protected |
| learn\_stepsize(double &epsilon, double adapt\_stat) | stan::mcmc::stepsize\_adaptation | inline |
| mu\_ | stan::mcmc::stepsize\_adaptation | protected |
| restart() | stan::mcmc::stepsize\_adaptation | inlinevirtual |
| s\_bar\_ | stan::mcmc::stepsize\_adaptation | protected |
| set\_delta(double d) | stan::mcmc::stepsize\_adaptation | inline |
| set\_gamma(double g) | stan::mcmc::stepsize\_adaptation | inline |
| set\_kappa(double k) | stan::mcmc::stepsize\_adaptation | inline |
| set\_mu(double m) | stan::mcmc::stepsize\_adaptation | inline |
| set\_t0(double t) | stan::mcmc::stepsize\_adaptation | inline |
| stepsize\_adaptation() | stan::mcmc::stepsize\_adaptation | inline |
| t0\_ | stan::mcmc::stepsize\_adaptation | protected |
| x\_bar\_ | stan::mcmc::stepsize\_adaptation | protected |


---

[ Stan Home Page ]

*© 2011–2014,
Stan Development Team.*
